# The influence of professional identity, job satisfaction, burnout on turnover intention among village public health service providers in China in the context of COVID-19: A cross-sectional study

**DOI:** 10.3389/fpubh.2022.925882

**Published:** 2022-09-20

**Authors:** Xuewen Zhang, Wenjie Zhang, Li Xue, Zongyou Xu, Zhuang Tian, Chao Wei, Ying Zhang, Zhihuan Dong, Shansong Gao

**Affiliations:** ^1^School of Integrated Traditional Chinese and Western Medicine, Jining Medical University, Jining, China; ^2^Department of Academic Affairs, West China School of Medicine/West China Hospital, Sichuan University, Chengdu, China; ^3^Medical Department of First Affiliated Hospital of Third Military Medical University, Southwest Hospital, Chongqing, China; ^4^Medical School, Hubei University for Nationalities, Enshi, China; ^5^School of Public Health, Jining Medical University, Jining, China; ^6^School of the Second Clinical, Jining Medical University, Jining, China; ^7^School of Clinical, Jining Medical University, Jining, China

**Keywords:** professional identity, job satisfaction, burnout, turnover intention, village public health service providers

## Abstract

**Background:**

In China, COVID-19 has undoubtedly posed a huge challenge to the capacity of rural public health services. Village public health service providers are responsible for reporting and dealing with infectious diseases and public health emergencies. However, the turnover of village public health service providers is gravely threatening the stability of rural primary health system step by step. This study systematically evaluated the effects of professional identity, job satisfaction, burnout on turnover intention of village public health service providers, and further measured the mediating effect of job satisfaction and burnout between professional identity and turnover intention.

**Methods:**

From May to June 2019, 1,244 village public health service providers in Shandong Province were selected as the research objects. Sociodemographic characteristics, professional identity, job satisfaction, burnout and turnover intention were quantitated by self-completed questionnaire and measured by Likert 5–7 scale. Person correlation analysis, One-way ANOVA, and Structural Equation Modeling (SEM) were used for statistical analysis and mediating effect evaluation.

**Results:**

Five hundred and sixty-four (45.3%) village public health providers had high turnover intension. Professional identity had a direct positive effect on job satisfaction (β = 0.146, *p* < 0.001), job satisfaction had a direct negative effect on burnout (β = 0.263, *p* < 0.001), and turnover intension (β = −0.453, *p* < 0.001), while burnout had a direct positive effect on turnover intension (β = 0.242, *p* < 0.001). Between professional identity and turnover intention, job satisfaction 95%CI: (−0.289)–(−0.11) had significant mediating effects. Job satisfaction 95%CI: (−0.216)–(−0.077) also had significant mediating effects between professional identity and burnout, and burnout had significant mediating effects between job satisfaction and turnover intension, 95%CI: (−0.116)–(−0.052). These results strongly confirm that professional identity, job satisfaction, and burnout are early and powerful predicators of turnover intention.

**Conclusion:**

According to the results, medical administration and management departments should pay attention to improve the professional attraction of public health services by improving the public's understanding of the profession, reducing work intensity, timely granting of subsidy funds, improving old-age security, ensuring the income level, increasing the new force and so on, so as to reduce turnover intension and ensure the health equity of village residents.

## Background

On January 2, 2021, after a 61-year-old woman was diagnosed with COVID-19 in Xiaoguozhuang Village, Hebei Province, China, hundreds of confirmed cases emerged in just a few days, fully exposing the weakest link in China's public health system in the vast village areas. As the focus and difficulty of national public health construction, the “rural three-level medical network” is composed of county-level medical and health institutions, township health centers and village clinics, which shoulder the important task of providing basic public health services for rural residents (1). Especially the village clinics are the first barrier for disease control and prevention in rural, in a large number of village population flow to cities today, if this barrier can't play an effective role or loopholes, it will pose a threat to the public security of the society, even bring catastrophic danger. Village public health providers, composed of village doctors in village clinics (not all village doctors undertake this work), provide village residents with 12 major items and 45 small items of basic public health services stipulated by the state, including vaccination, health education and the establishment of health records etc., and they also undertake primary diagnosis and treatment of common and frequent diseases of village residents, which makes them play an important role in achieving equal access to basic public health services and safeguarding the health rights and interests of village residents (2).

After the implementation of the new medical reform policy aimed at strengthening the grass-roots level in 2009, the service capacity of village public health providers has become more important. On the one hand, it directly affects the success or failure of the national basic public health service projects, and on the other hand, it directly affects the health of village people, who account for about half of the national population. However, it has been proved that the investment in basic public health services in rural areas only accounts for about 1/4 of that in cities, which makes the per capita public health resources in village areas seriously inadequate, especially full-time public health human resources (3). This special situation makes village doctors become the backbone to guarantee basic public health services in village areas.

Excellent village public health providers can improve the quality of public health services in village areas. However, due to the heavy health work, poor living environment, low income, inadequate compensation for basic public health services, and lack of implementation of the pension system (4), many excellent village public health providers are unwilling to engage in basic public health services, or even leave their posts as village doctors (5). In recent years, village doctors in Henan and Gansu provinces of China have collectively left their jobs due to inadequate compensation for basic public health services, which leads to the lack of excellent health service providers and the inability to provide good health services for village residents (6). Some regions have reduced the qualification standards for doctors to compensate for the shortage of public health service providers, so that some people with imperfect or incomplete health skills can serve as health service providers, which leads to the situation of three low levels (low educational background, low ability and low quality of service) in the village public health providers, and further aggravates the problems existing in the construction of basic public health service system in rural areas (5), and the ability of village emergency response to public health emergencies.

COVID-19 has undoubtedly posed a huge challenge to the capacity of village public health services. In January 2021, there were multiple clusters of outbreaks in village areas of Hebei province, and the recent outbreak in Henan province also started from village areas, which not only sounded the alarm bell for village epidemic prevention and control, but also exposed that village public health management has become a weak board in epidemic prevention and control. As the “health gatekeeper” of village residents, village public health providers are responsible for reporting and handling infectious diseases and public health emergencies, and their ability to provide emergency public health services will directly affect the physical and mental health and safety of village residents, as well as the spread and expansion of epidemic diseases (7). It can be seen that village public health providers' recognition of their profession, work satisfaction and burnout may not only affect their turnover intention and the stability of the team, but also affect the solid foundation of the regular epidemic prevention and control, as well as the life and property safety of village residents (6).

Turnover intention of employee has become one of the important topics in organizational research, and many scholars even believe that it is more meaningful to study turnover intention than the actual turnover behavior (8, 9). First, actual turnover behavior is affected by many external factors and is more difficult to predict than turnover intention. In view of this, Bluedorn (8), Price and Mueller (10) even suggested that turnover intention should replace actual turnover behavior in the study. Secondly, the turnover of the organization is the change in dynamic over time, and the study begins with different times, and the conclusions can be different (11). Thirdly, consistent with attitude, desire and behavior, turnover intention is usually regarded as the most direct “predictor” of turnover behavior and there is a very significant direct correlation between them, with the correlation coefficient reaching 0.50 (12).

In recent years, there have been many studies on turnover intension of medical staff, and the influencing factors include personal factors, organizational factors, and environmental factors. A systematic review involving six studies showed that factors affecting nurses' turnover intention were divided into individual factors and organizational factors, among which job satisfaction was the most important factor (13). A survey of 327 medical staff in Taiwan, China, showed that workload and job burnout both had a significant positive impact on turnover intention, leading to a high turnover rate of medical staff and a decline in medical quality (14). A survey of 1,152 primary medical staff in Anhui Province, China, showed that age, job position and burnout affected the correlation of turnover intention, psychological capital and social support were negatively correlated with turnover intention, and job burnout was positively correlated with turnover intention (15).

At present, the research on turnover intension of public health service providers is less than that of doctors, and the research on village public health providers is even rarer. A study of 3,212 urban community public health workers in five Chinese provinces found that 38.7% of them had an intention to leave their jobs. The influencing factors include: age, salary, post, professional title, learning and training opportunities, work pressure, etc. (16). Among 926 public health service providers in Shanghai, 42.44% had turnover intention. Vitality, dedication, depersonalization and low sense of personal accomplishment had significant predictive effects on turnover intention (17). In June 2018, 25.23% of the 436 primary medical staff engaged in the national basic public health service projects in Guangzhou had the intention to leave, the risk factors were high work pressure and the number of national basic public health service projects in charge ≥3 (18). In Thailand, more and more grass-roots public health workers are moving from village public health services to urban private hospitals, putting the village public service system at risk (19). 77.3% of doctors in Iran's Village Family Health Doctor Program, which began in 2005, plan to quit in the near future. Rare opportunities for continuing education, inappropriate and long working hours, irregular pay, lack of job security and high level of job responsibility were cited as the most important reasons (20). The Philippines has implemented the 'Doctors to The Barrios' (DTTB) Program for public health services for remote village populations. Participants joined the project out of interest in public health and a desire to serve rural residents, but a significant decrease in job satisfaction, salary, and development influenced participants to accelerate turnover (21). An American study found that the inability to recruit well-trained providers, low salary grade, aging labor force synergically affected the development of village public health service programs (22).

In the context of COVID-19, academic researches on medical staff mainly focus on the psychological status (23), psychological crisis (24), psychological intervention (3), job burnout (25), new positioning of professional identity, as well as knowledge and countermeasures of epidemic prevention and control of staff in different departments (26). For example, Wu Xuefen evaluated the mental health of 813 grass-roots medical staff in Shanghai and found that during the new crown epidemic, medical staff were more fatigued, excessively worried, insomnia, and troubled (27). During COVID-19, Zhang Xiaoyan et al. measured the work pressure of 615 medical staff in 13 community health service centers in Wuhan and found that the work pressure of primary medical staff was very high, and was negatively correlated with the ability to respond to public health emergencies (28). Lei and Li found that 24.9% of community health service providers experienced psychological anxiety during COVID-19, job satisfaction and worry about inadequate reserve of protective materials were the factors that affect the psychological anxiety of them (29). However, the research on rural grassroots medical staff during the COVID-19 epidemic is relatively limited, and the research on rural public health services is even rarer in the academic circle. There are few studies on the working status of village public health service providers during the COVID-19 epidemic, especially turnover intention. Existing research mainly focused on the staff's age, salary, training, work environment and mostly use chi-square test, *T*-test, analysis of variance (ANOVA), multiple linear or multiple logistic regression analysis and other methods to analyze the working conditions and influencing factors of village public health service providers (13, 18–24). Compared with the above studies, the structural equation model (SEM) can not only measure the correlation between the study variables, but also mine the correlation between potential variables, and even explain the causal relationship between variables.

Professional identity is a second-order structure composed of four dimensions: professional practice, professional affirmation, commitment identification and commitment reflection. Many studies on doctors and nurses (30–32) have confirmed that professional identity had a negative impact on burnout and turnover intention and a positive impact on work engagement and satisfaction, and can indirectly affect burnout and turnover intention through these two mediating variables. Lack of professional identity leads to higher rates of job burnout and turnover intension, which affect both individuals and organizations. Moreover, an in-depth study had confirmed that professional identity was one of the strong predictors of the turnover of public health service supervisors in village China, and was an intermediary factor between job satisfaction, burnout, and turnover intention. It is suggested that the Chinese government can reduce the turnover intention of health inspectors by improving professional identity (33).

As another strong predictor of turnover intention, many medical occupational studies have confirmed that job satisfaction is strongly correlated with turnover intention, and it can also affect turnover intention and behavior through the mediating effect of other job-related factors (34). For example, there is a correlation between job satisfaction and organizational commitment, and the higher the job satisfaction, the higher the organizational commitment. On the contrary, there is a high reverse correlation between organizational commitment and turnover intension, which should be reduced by improving job satisfaction that affects organizational commitment (35). Another study confirmed that the hospital can improve nurses' job satisfaction by guiding them to pursue higher levels of work values, so as to minimize nurses' turnover intension and turnover rate (36). A study on rural health human resources in China strongly confirmed, as an early and strong predictor of turnover intention, job satisfaction of village doctors not only had a direct negative impact on turnover intention, but also had an indirect impact on turnover intention through work engagement (5).

Maslach and Jackson's three-dimensional view divided burnout into three core components, namely “emotional exhaustion, deindividuation and low personal achievement”. Among them, emotional exhaustion refers to the excessive consumption of individual emotional resources, emotional exhaustion, and complete loss of work enthusiasm. Deindividuation refers to a negative, indifferent, overly distant attitude toward service objects. Low sense of personal achievement refers to the individual's decreased sense of competence and work achievement, and the tendency to negatively evaluate the meaning and value of their own work (37). Many studies have confirmed that job burnout is positively correlated with turnover intention (28), and job burnout significantly reduces the job satisfaction of medical staff and increases the turnover intention of them (29). Compassion fatigue of nurses will lead to burnout and thus increase the turnover intension (30). The stronger the psychological pressure of nurses, the stronger the job burnout, the lower the job satisfaction and the stronger the turnover intension (31). Therefore, humanistic management of nursing staff can effectively prevent the occurrence of job burnout and reduce the turnover intension (32).

**Figure 1:**
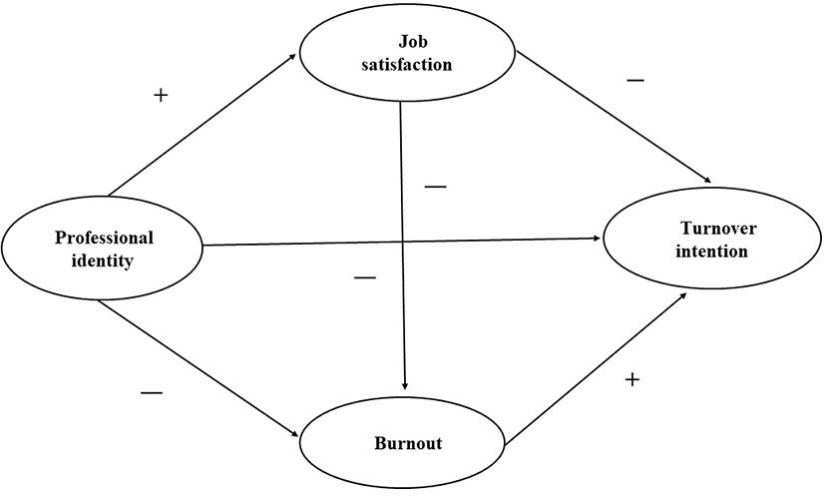
The theoretical model and hypotheses.

COVID-19 is a huge challenge to the capacity of village public health services and an opportunity to enhance the capacity (38). Therefore, based on the above theories and literature conclusions, this study for the first time tries to propose the hypotheses of professional identity, job satisfaction, burnout, turnover intention, and constructs the dual intermediary data model shown in Table 1 and Figure 1. We hypothesized that professional identity, job satisfaction and burnout of village public health service providers have a direct impact on turnover intention. In addition, professional identity also indirectly affects turnover intention through the mediating effects of job satisfaction and burnout respectively. The results of this study will provide evidence support for improving the professional identity and job satisfaction of village public health service providers, solving their income and security problems, reducing turnover intension, improving the stability and work efficiency of the team, and enhancing their ability to prevent and control the epidemic. It also provides suggestions and opinions for establishing a comprehensive rural public health emergency system (38).

**Table 1:** The theoretical hypotheses.

**Hypotheses**
1. Village public health service providers' **professional identity** has a negative impact on **turnover intention**.
2. Village public health service providers' **job satisfaction** has a negative impact on **turnover intention**.
3. Village public health service providers' **burnout** has a positive impact on **turnover intention**.
4. Village public health service providers' **professional identity** has an indirect negative impact on **turnover intention** through the mediating effect of **job satisfaction**.
5. Village public health service providers' **professional identity** has an indirect negative impact on **turnover intention** through the mediating effect of burnout.
6. Village public health service providers' **professional identity** has an indirect negative impact on **burnout** through the mediating effect of **job satisfaction**.
7. Village public health service providers' **job satisfaction** has an indirect negative impact on **turnover intention** through the mediating effect of **burnout**.

## Methods

### Setting and participants

Jining is in the inland southwest of Shandong Province, and the level of economic development lags several coastal cities in the east, especially in rural areas. The total population of the city is 8.356 million, among which the rural population is 3.3683 million, accounting for 40.3%. There are 6,489 villages and 5,307 village clinics serving the health of village residents. According to the statistical bulletin, by the end of 2018, the total number of medical providers in the city was 80,800, an increase of 2,100 compared with 2017, but the increase of medical providers was mainly concentrated in cities, while the number of village doctors was only 11,715, a decrease of 870 compared with 2017 (39). The average number of village doctors in each village was <2 (1.81), but the number of patients diagnosed and treated by village clinics in a year was far more than 20 million, with an average of 1,814.94 patients treated by each village doctor per year. While completing the daily clinical treatment, the basic public health services of village residents are also taken into account by some village doctors. Therefore, the work is heavy and the current situation of turnover is not ignored. In view of the above reasons, it is of great significance to select Jining City as the sample location to understand the turnover intension and influencing factors of village public health service providers.

With the assistance of Municipal Health and Health Commission, multi-stage stratified cluster random sampling method was used to select research objects. Firstly, 11 counties (districts) in Jining were divided into good, medium, and poor grades according to the level of economic development. Secondly, one county of each of the three grades was selected as the sample point of this study through simple random sampling method. Thirdly, all village doctors in the sample counties (districts) were selected by cluster random sampling. Finally, part-time basic public health service providers were selected as the final research objects of this study.

As the research objects were village doctors with a certain level of education and understanding, data were collected through on-site collective distribution and self-filling questionnaires, including cover letters, general sociological characteristics, and scales. In order to reduce the error and improve the recovery rate, we first explained the significance of the study in detail in the cover letter and the protection of personal information for anonymous survey, thus improving the response rate. Second, assigned special providers at the city, county, township, and village levels to be responsible for the distribution, recovery, and quality verification of questionnaires to ensure the quantity and quality of questionnaires.

In this study, 2,789 village doctors from 1,345 village clinics were included in the survey, 2,693 questionnaires were collected, of which 2,684 were valid, with an effective rate of 96.2%. Among 2,684 village doctors, 1,244 were engaged in public health services, so the final research object of this study was 1,244 village public health service providers.

### Ethical and participatory consent

Our research group applied for ethical review to the Faculty Review Committee. The academic panel considered that since the study did not involve medical human-biological interventions, and the data presented in the paper could not be traced back to individuals, so the study could collect data based on participants' verbal consent. Before the on-site questionnaire survey, we introduced the purpose and significance of this study to all participants, and informed them that their participation was voluntary and anonymous, and the statistical data produced by the study could not be traced to individuals. They had the right to refuse to participate in the survey or terminate the survey at any time, and the questionnaire completed by the exiting investigator was destroyed on the spot.

### Measures

According to China's national health committee of the human resources for health questionnaire (6), the questionnaire consists of five parts, the first part: the survey of the population characteristics (age, gender, education level, and marital status) and job characteristics (professional, job title, salary, working years, and average weekly working hours). The second to fifth parts are scales of job satisfaction, professional identity, burnout, turnover intension, etc.

#### Professional identity

Professional identity is the basis for the normal functioning of professional roles. Studies have shown that nurses with better professional identity are more flexible in implementing professional role changes, which is reflected in best nursing practices and patients' health maintenance benefits (40). The Chinese version of the professional identity questionnaire includes three items: (1) My work has significant significance and influence on other people's lives; (2) The quality and effect of my work will affect many people; (3) My work is very meaningful and important. All these items were assessed on a five-point Likert scale 1 (highly disagree) to 5 (highly agree), with a higher score indicating a higher degree of agreement with the significance of the work undertaken. After verification by many studies, this questionnaire has good reliability and validity, and Cronbach's Alpha coefficient of this questionnaire is 0.831 (41).

#### Job satisfaction

Job satisfaction is an important psychological indicator of employees' professional life quality. If a person's job satisfaction level is high, he or she may be more positive about work, and vice versa. In this study, the Chinese version of Job Description Index scale with Cronbach's Alpha coefficient of 0.951 (42), which has been verified for many times, was used to measure the job satisfaction of village public health providers. It includes eight items: workload, colleagues, superiors, environment and facility, promotion, income, social security, training opportunities (6). All these items were assessed on a six-point Likert scale from 1 (highly disagree) to 6 (highly agree), with higher scores associated with higher job satisfaction and more likely to be positive about work.

#### Burnout

The Chinese version of Maslach Burnout Scale general scale (MBI-GS) was used to measure the burnout of village public health service providers, and the Cronbach's alpha value was 0.79–0.94 (43, 44). The scale includes three subscales: emotional exhaustion, depersonalization, and low personal achievement (reverse score). The answer is set to a 7 Likert score ranging from 0 (never) to 6 (daily). Among them, emotional exhaustion scores >25, depersonalization scores >11, low personal achievement scores >16 are defined as high burnout, respectively. Burnout can be diagnosed when the score of any dimension of the respondents is greater than the critical value (45).

#### Turnover intention

Chinese version of turnover intention questionnaire prepared by Cammannet and Mobley (33) was used to measure the turnover intention of the respondents. The scale has been modified for many times and perfected in Chinese, and the Cronbach alpha coefficient is 0.659 (46). It consists of four items: “Thought of leaving the organization you serve now,” “Thought of leaving this industry,” “Looking for a new job recently,” “Looking for a new job next year”. A six-point Likert scale ranging from 1 (highly disagree) to 6 (highly agree) was used to assess all of these items, with higher scores and higher turnover intentions (6).

### Statistical analysis

The reliability and validity of the whole questionnaire were evaluated scientifically and accurately by exploratory factor analysis (EFA). The demographic characteristics and job-related characteristics of 1,244 village public health service providers were classified and described by descriptive statistical analysis method. Then, professional identity, job satisfaction, burnout and turnover intention were quantitatively analyzed, and the statistical results were presented as mean and standard deviation (SD). Pair-based correlations between the values of the main variables were measured by Pearson correlation and quantified by correlation coefficients. On this basis, a more scientific Structural Equation Model (SEM) was used to further explore the relationships among the four dimensions of professional identity, job satisfaction, burnout and turnover intention, and the maximum likelihood model based on Bootstrap was applied to SEM. Several key indexes of fit degree of measurement model and data, including goodness of fit index (GFI), standard fit index (NFI), Comparative fit index (CFI), Adjusted goodness of Fit index (AGFI), Tuck-Lewis index (TLI) and incremental index (IFI), were all >0.90. The approximate root mean square error (RMSEA) is 0.067, lower than 0.8, which turns out to be an acceptable model and fits the current data and assumptions. Bootstrap sampling method was used to test the mediating effect, and the existence of the mediating effect was judged according to whether the 95% confidence interval of the product term (a^*^b) of regression coefficient A and regression coefficient B included the number 0. If the 95% confidence interval does not include the number 0, the mediating effect is indicated and if the 95% confidence interval includes the number 0, there is no mediating effect.

### Reliability and validity

According to EFA results, Kaiser-Meyer-Olkin (KMO) of the questionnaire was 0.874, >0.70, and Bartlett sphericity test showed significant difference (χ^2^ = 15,223.125, *P* < 0.001), all which indicating a good possibility of data factor analysis (33, 47–49). The Cronbach's α-values of job satisfaction, turnover intention, job burnout and professional identity are as high as 0.907, 0.957, 0.834, and 0.831, all >0.7, indicating that the sample data has good reliability (50, 51). The Average Variance Extracted value of the mean variance extraction of each dimension is >0.5, indicating that each dimension of the measurement model has good Convergence Validity (51) (Table 2). Discriminant Validity can be used to accurately assess the degree of unique difference between a particular structure and other structures in the model (52). Discriminant validity was tested by comparing the square root of AVE for individual constructs with the correlations among the latent variables. By comparing the square root of AVE in **Table 5** with all correlation coefficients, a reliable discriminant validity can be established when diagonal elements exceed off-diagonal elements (53, 54).

**Table 2:** Reliability and validity test.

**Dimension**	**Item**	**Unstd**.	**S.E**.	***Z***	***P***	**Std**.	**Cronbach's α**	**SMC**	**CR**	**AVE**
Turnover intension	T1	1.00				0.92	0.96	0.85	0.95	0.84
	T2	1.07	0.02	67.39	***	0.97		0.94		
	T3	1.02	0.02	56.19	***	0.91		0.84		
	T4	0.91	0.02	45.18	***	0.84		0.71		
Professional	P1	1.00				0.76	0.83	0.58	0.84	0.64
	P2	1.41	0.06	23.92	***	0.93		0.87		
	P3	1.23	0.05	24.17	***	0.69		0.47		
Burnout	B3	1.00				0.86	0.83	0.74	0.83	0.63
	B2	0.96	0.03	28.41	***	0.87		0.76		
	B1	0.58	0.03	21.92	***	0.61		0.37		
Job satisfaction	J1	1.00				0.77	0.91	0.59	0.90	0.54
	J2	0.61	0.03	18.21	***	0.52		0.27		
	J3	1.03	0.04	28.84	***	0.79		0.62		
	J4	0.72	0.03	22.20	***	0.63		0.39		
	J5	0.96	0.03	27.76	***	0.76		0.58		
	J6	1.07	0.04	30.22	***	0.82		0.67		
	J7	0.85	0.03	25.54	***	0.71		0.50		
	J8	1.10	0.04	31.28	***	0.84		0.71		

*** indicates *P* values less than 0.001.

## Results

### Demographic characteristics of participants

Demographic characteristics of 1,244 village public health service providers are shown in Table 3. The average age was (45.3 ± 7.2) years old, and was mainly concentrated in the 40–49 years old group, accounting for 49.3. While only 0.6% were under 30 years old, and 27.4% were 50 years old and above. 66.3% of the respondents had only technical secondary education, while only 3.1% had university or higher education, 3.4% had intermediate or senior professional titles, and 40.9% had no title. Forty-two percent have worked between the ages of 20 and 29, 52.8% have a monthly income of <2,000 Yuan, and 72.4% must work 60 h or more per week.

**Table 3:** Demographic characteristics of participants (*n* = 1,244).

**Socio–demographic**	***N***	**%**
**Gender**		
Male	826	66.4
Female	397	31.9
Missing	21	1.7
**Age, group**		
<30 years	7	0.6
30–39 years	263	21.1
40–49 years	613	49.3
≥50 years	341	27.4
Missing	20	1.6
**Professional ranks**		
Senior title	4	0.3
Mid-level title	38	3.1
Primary title	619	49.8
No title	509	40.9
Missing	74	5.9
**Years of work**		
<10	26	2.1
10–19	404	32.5
20–29	522	42.0
≥30	255	20.5
Missing	37	3.0
**Marital status**		
Unmarried	27	2.2
Married	1,171	94.1
Missing	46	3.7
**Education background**		
University or above	39	3.1
Junior college	312	25.1
Technical secondary school	825	66.3
High school education or below	49	3.9
Missing	19	1.5
**Monthly income (yuan)** ^*^		
<1,000	196	15.8
1,000–1,999	460	37.0
2,000–2,999	321	25.8
≥3,000	179	14.4
Missing	88	7.1
**Weekly working hours**		
<40	161	12.9
40–59	129	10.4
≥60	905	72.8
Missing	49	3.9

* As of the date of this paper writing, the exchange yuan-euro exchange rate according to the People's Bank of China was 0.1278.

### Descriptive analysis of study variable

The total scores of job satisfaction, professional identity, burnout, and turnover intention were 31.56 ± 9.39, 9.27 ± 1.98, 42.29 ± 21.03, 11.91 ± 6.18, respectively. The sub-dimensional statistical results contained in each indicator are shown in Table 4, respectively. In terms of scores, 564 (45.3%) village public health providers had high turnover intension, 347 (27.9%) had medium, and 333 (26.8%) had low turnover intension. Work load (3.72 ± 1.57), social security (3.45 ± 1.56), promotion (3.66 ± 1.56), and income (3.17 ± 1.57), were lower than other items of job satisfaction, and “Thought of leaving the organization you serve now” (3.06 ± 1.63) and “Thought of leaving this industry” (3.05 ± 1.66) were higher than other items of turnover intention.

**Table 4:** Item scores in job satisfaction, professional identity, job burnout, and turnover intention.

**Items**	**Mean ±SD**
**Job satisfaction**	31.56 ± 9.39
Workload	3.72 ± 1.57
Colleagues	4.67 ± 1.40
Superiors	4.65 ± 1.37
Environment and facility	4.02 ± 1.51
Promotion	3.66 ± 1.56
Income	3.17 ± 1.57
Social security	3.45 ± 1.56
Training opportunities	4.19 ± 144
**Professional identity**	9.27 ± 1.98
My work has a great impact on the lives of others	2.98 ± 0.82
The quality of my work will affect many people	3.10 ± 0.76
My work is very meaningful and important	3.20 ± 0.70
**Burnout**	42.29 ± 21.03
Emotional exhaustion	18.64 ± 12.43
Low personal achievement	18.01 ± 13.82
Depersonalization	5.64 ± 6.87
**Turnover intention**	11.91 ± 6.18
Thought of leaving the organization you serve now	3.06 ± 1.63
Thought of leaving this industry	3.05 ± 1.66
Looking for a new job recently	2.99 ± 1.67
Looking for a new job next year	2.82 ± 1.61

### Correlations of study variables

The Pearson correlation coefficients of the four main observed variables of village public health service providers are shown in Table 5. Professional identity is positively correlated with job satisfaction, and negatively correlated with burnout and turnover intention. Finally, burnout is positively correlated with turnover intention.

**Table 5:** Correlation and discriminant validity between potential variables.

**Item**	**Professional identity**	**Job satisfaction**	**Burnout**	**Turnover intension**
Professional identity	**0.80** *			
Job satisfaction	0.19	**0.74** *		
Burnout	−0.24	−0.30	**0.79** *	
Turnover intension	−0.09	−0.46	0.33	**0.91** *

* The bold diagonal elements are the square roots of each AVE: construct correlations are shown off-diagonal.

### Testing of the constructed study model

**Figure 2:**
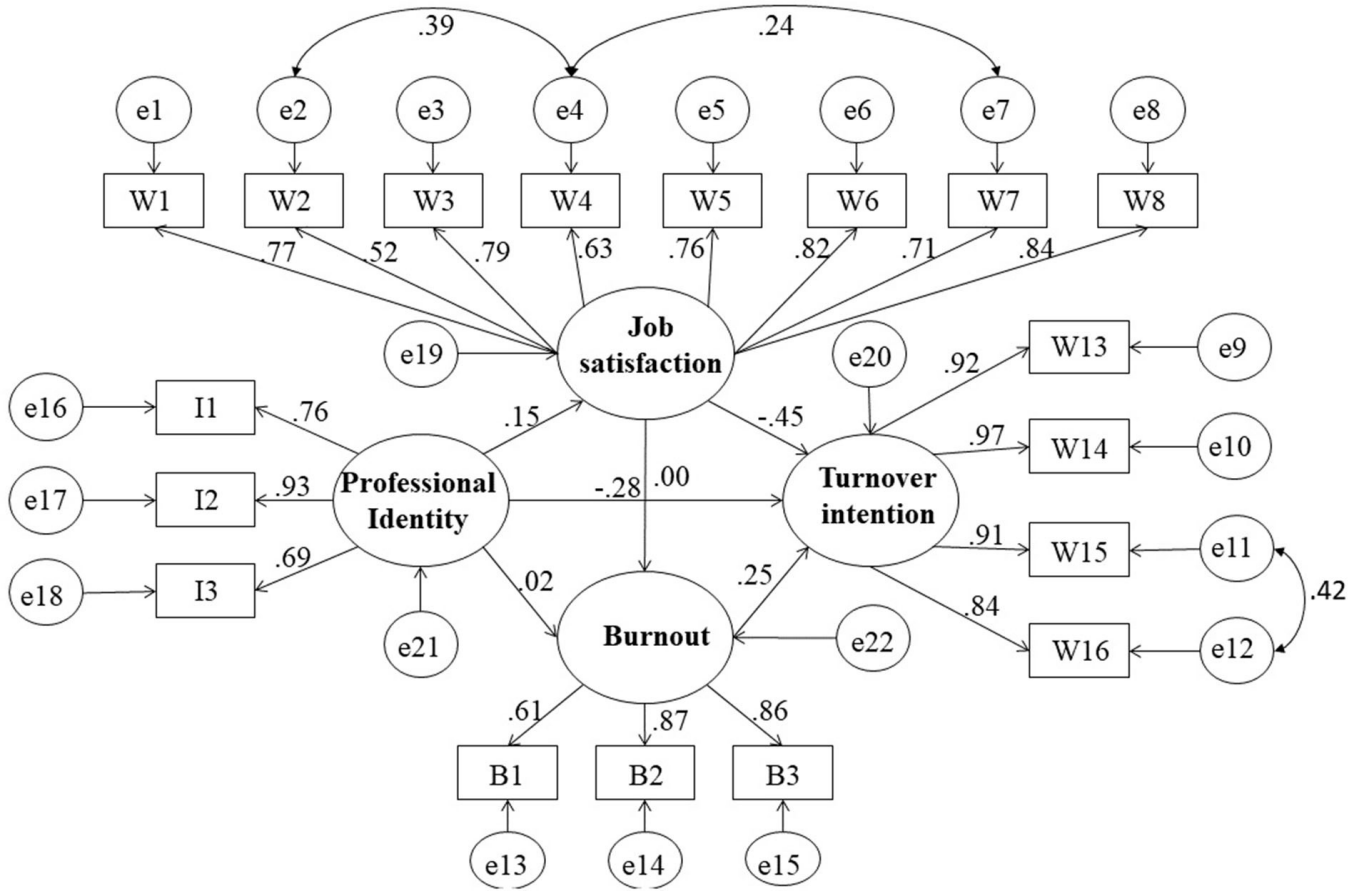
The final model and standardized model paths.

In the establishment of the structural equation model, including gender, age, income and other demographic and job-related characteristics, which are not predictive factors, but may have a mixed effect on turnover intention, therefore, we put these 8 factors (Table 3) into the equation and add their paths to the turnover intention of the result variable. In this way, the relationship between the effects of the predicted variables (job satisfaction, professional identity, and job burnout) on the outcome variable (turnover intention) can be linked, quantified and evaluated after accurately controlling these variables (55). The generalized least square method was used to fit the data into the theoretical model constructed before, and the model was modified and improved according to the model fitting index. Finally, the established model indicates the relationship between the four variables and the path validity (Figure 2). The fitting indexes of the final modified hypothesis model were NFI = 0.931, RFI = 0.922, IFI = 0.948, TLI = 0.941, CFI = 0.948, and RMSEA = 0.048, all of which met the requirements of reference value and the model fit was good.

Maximum likelihood estimation was adopted, and each path was repeatedly guided by 200 bias correction bootstraps. The mediation analysis path and effect values are shown in Table 6. Professional identity had a direct positive effect on job satisfaction (β = 0.146, *p* < 0.001), job Satisfaction had a direct negative effect on burnout (β = −0.263, *p* < 0.001), and turnover intension (β = −0.453, *p* < 0.001), while burnout had a direct and significant positive effect on turnover intension (β = 0.242, *p* < 0.001).

**Table 6:** Significance test of the mediating test.

**Model pathways**	**Estimated**	**95%CI**
**Total effects**		
Job satisfaction ← Professional identity	0.146	0.069–0.219
Burnout ← Professional identity	−0.016	(−0.083)−0.051
Turnover intension ← Professional identity	−0.070	(−0.134)–(−0.006)
Burnout ← Job satisfaction	−0.263	(−0.330)–(−0.197)
Turnover intension ← Job satisfaction	−0.517	(−0.570)–(−0.460)
Turnover intension ← Burnout	0.242	0.182–0.299
**Direct effects**		
Job satisfaction ← Professional identity	0.146	0.076-−0.220
Burnout ← Professional identity	0.023	(−0.043)−0.091
Turnover intension ← Professional identity	0.000	(−0.053)−0.053
Burnout ← Job satisfaction	−0.263	(−0.326)–(−0.199)
Turnover intension ← Job satisfaction	−0.453	(−0.154)–(−0.394)
Turnover intension ← Burnout	0.242	0.182–0.299
**Indirect effects**		
Burnout ← Professional identity	−0.038	(−0.061)–(−0.018)
Turnover intension ← Professional identity	−0.070	(−0.114)–(−0.027)
Turnover intension ← Job satisfaction	−0.064	(−0.087)–(−0.043)

Table 7 displays the significant test results of the two mediated pathways. In the intermediary path between professional identity and turnover intention, job satisfaction 95%CI: (−0.289)–(−0.11) had significant mediating effects. Job satisfaction 95%CI: (−0.216)–(−0.077) also had significant mediating effects between professional identity and burnout, and burnout had significant mediating effects between job satisfaction and turnover intension, 95%CI: (−0.116)–(−0.052).

**Table 7:** Significance test of every mediating pathway.

**Model pathways**	**95%CI**
Turnover intention ← Job satisfaction ← Professional identity	(−0.289)–(−0.111)
Burnout ← Job satisfaction ← Professional identity	(−0.216)–(−0.077)
Turnover intension ← Burnout ← Job satisfaction	(−0.116)–(−0.052)

## Discussion

The COVID-19 outbreak is raging and developing rapidly, and China is joining forces from top to bottom to fight the virus. As the “health gatekeeper” of village residents, village public health service providers are the bottom of the health service system, playing an important fundamental role in volunteer screening, temperature monitoring, publicity and education. The important and unique value of this study lies not only in the fact that we selected public health service providers in rural China who are at the forefront of the fight against COVID-19 as research objects, but also in the fact that the four variables of job satisfaction, professional identity, burnout, and turnover intention are included into the structural equation model for the first time. On this basis, some suggestions are put forward to improve the village public health emergency system.

Both domestic and foreign epidemic prevention and control shows that villages are the first line of defense for village epidemic prevention and control, and village public health service providers always stand at the forefront to fight against the virus. The total score of turnover intention of village public health providers is 11.91 ± 6.18, was higher than the scores of township health inspectors in China (10.22 ± 5.49), and the scores of the four sub-dimensions were also higher than the latter (23). In terms of proportion distribution, the high turnover intention of village public health service providers (45.3%) is not only significantly higher than that of China's 1,109 urban community health service providers (4.3%) (56), but also higher than that of public health service providers in township health centers (35.1%) (57), and township health inspectors (11.3%) (23). It can be seen that the intention of village public health providers to quit their jobs is relatively severe. Although the survey time of this study was In May 2019, half a year earlier than the outbreak of the COVID-19 epidemic, it can be inferred from the data that the employment status of village public health service providers is not optimistic.

The results of structural equation model show that professional identity of village public health service providers has no direct influence on turnover intention and job burnout, but mediates through job satisfaction. At present, the research on the professional identity, turnover and burnout of medical personnel is mostly focused on the nurses in the oncology or emergency departments of the hospital, and it is concluded that there is a negative correlation between professional identity, job burnout and turnover intention (58), and the study of village public health service providers is relatively rare, the reason is that in the rural health service system in China, rural basic public health services do not have full-time staff, the village doctors concurrently engaged in public health services. The professional identity of village public health providers can be understood as their self-judgment on their knowledge and understanding of public health services, and is the recognition and inner acceptance of the role of public health given by the society. Some scholars believe that village public health service providers have a large demand gap but low salary and professional identity, so it is imperative to strengthen the construction of the talent team (59). The pension insurance problem of village public service providers has not been solved for a long time, which fundamentally affects their identity (60). The COVID-19 epidemic has made the whole society, including the village public health service providers, realize the importance of public health, and their professional identity during the epidemic is significantly better than that before the epidemic. Participating in epidemic prevention and control work to protect the life safety of rural residents makes them tend to have more positive self-evaluation and professional identity (61). Thus, it can be seen, the professional identity of village public health providers can withstand the test of the epidemic, while some mediating factors affect their high burnout and turnover intension. How these mediating factors play a role in the relationship between professional identity and turnover intension, as well as how to prevent and control them need to be further studied and explored.

At present, the academic investigation on the job satisfaction of public health service providers mostly focuses on the staff of urban community health service centers and township health centers, while the investigation of the most basic village public health service providers is very insufficient. For example, in 2019, a study covering 553 cases of basic public health service providers in Zhejiang province, Shanxi Province and Chongqing municipality found that 39.5% of employees were dissatisfied with their overall work. Social respect, performance appraisal, work ability and work intensity are the important factors affecting overall job satisfaction (62). In order to improve the equality of public health services in rural areas, China has implemented a rural cooperation mechanism for service projects. Township health centers are the main body undertaking service projects and village health centers assist in completing projects and accept the guidance and assessment of township health centers. The quality of public health service can be effectively improved only when they have clear division of labor and cooperate with each other. That is not the case, however, a covering 365 village public health service provider's investigation reveals that the project responsibilities unclear, large work volume on health services, funds allocation is unreasonable, even be diverted, lack of effective training has seriously affected the job satisfaction of village public health service providers and their enthusiasm to participate in rural collaboration (63). In 2019, in many rural areas, due to insufficient public health funds and the withholding of public health subsidies from public health providers, causing them to leave their jobs collectively, and some villages could not find a single village doctor or public health service provider. Village public health service providers are the most important force and professionals at the forefront of COVID-19 prevention and control. How to improve their job satisfaction and enthusiasm, reduce turnover and stabilize the ranks has become a key issue concerning the success of epidemic prevention and control.

In this study, burnout not only has a direct positive effect on the turnover intention of village public health service providers, but also plays a mediating role in the negative effect of job satisfaction on the turnover intention. Job burnout is mainly manifested in treating work negatively and neglectfully, underestimating own work value, and treating others with indifference. When the employee's job burnout accumulates continuously, it may eventually lead to his/her resignation. The overall level of job burnout of emergency department nursing staff was relatively high, and their turnover intention was relatively strong (64). The burnout scores of village public health service providers in this study are similar to the burnout scores of village doctors in our previous study. However, among the three dimensions, The score of low sense of accomplishment of village public health service providers was 18.01 ± 13.823, which was slightly higher than that of village doctors (17.53 ± 13.419) (45), which meant that village public health service providers had a low sense of accomplishment. Many relevant studies show that the main factors affecting the low sense of achievement of basic public health service providers include annual income level, continuing education situation, uncooperative service objects, heavy workload, promotion space of the unit, health status, lack of family support and other factors (33, 45, 65, 66). Combined with the results of job satisfaction scale analysis in this study, it is not difficult to find that among the village-level public health service providers surveyed, the lowest score of job satisfaction was income 3.17 ± 1.57, followed by social security 3.45 ± 1.56, promotions 3.66 ± 1.56 and workload 3.72 ± 1.57. The low sense of achievement leads to the increase of burnout, which may eventually accumulate to a certain extent and cause resignation.

To sum up, the structural equation is used in this study to reveal the three influencing pathways of village public health provider's turnover intention, and it is confirmed that professional identity, job satisfaction and burnout are all accurate predictors of village-level public health provider's turnover. The results further verified the complex influence of professional identity on turnover intention, which is complicated because professional itself has no direct negative impact on turnover intention, but can indirectly affect turnover intention through the mediating effect of job satisfaction and burnout.

Two limitations should be considered in this study, first of all, although SEM is used in this study to test the complex relationship between variables, the design type of this study is a cross-sectional study, that is, at the beginning of the study, factors as “causes”, including professional identity, job satisfaction and burnout, and factors as “outcomes”, that is, turnover intention exist simultaneously, therefore, the sequence of cause and outcome factors could not be determined, and no control group was established for comparison and identification. Therefore, confirmatory conclusions of causality among factors cannot be determined based on cross-sectional design. Secondly, we collect data through questionnaires filled out by the respondents themselves instead of strict face-to-face survey, which may be unable to avoid the biased influence of subjective differences of the subjects on data quality.

## Conclusions

The high turnover rate of village public health service providers is costly for the rural public health system, the epidemic prevention and control system, and rural residents, and will lead to an imminent passive situation in the rural response to major public health emergencies such as COVID-19. To ensure a high degree of “fit” between work and individual is an important guarantee to reduce the turnover rate.

In the absence of major epidemics or other public health crises, people generally do not pay enough attention to the public health profession, and there is a phenomenon of “emphasizing clinical practice over prevention”. The professional identity of village public health providers should be strengthened and cultivated at the stage of medical education (67, 68), however, at present most of the research in public health education more focus on the pros and cons of China's public health professional education system, and the lack of research on public health professional students' professional identity (69), many students, especially students in lower grades for their professional sciolistic, lack of interest in learning, which also buried hidden trouble for his turnover after work (70).

Therefore, in the context of COVID-19, the government should strengthen the popularization of professional knowledge of public health to improve public awareness, especially for high school students, to increase their enthusiasm to apply for the major. At the same time, the medical colleges and universities should carry out pre-conceived professional education and guidance when the public health students enter the school, so that they can understand the nature, content, and far-reaching significance of the future work in advance (69). It is more necessary for schools to improve training programs, conduct in-depth studies on occupational and industrial standards of public health majors, and optimize curriculum setting and training mode from the perspective of job tasks, to improve the learning enthusiasm of students majoring in public health and improve their recognition of the profession after employment, then reduce burnout and turnover at work, and truly become the public health talents in line with the rural grassroots medical and health service system (69).

Government and society should pay attention to the development of village health service provider, improve their job satisfaction through multiple ways and channels, and influence their job prospects identity and work embedded degrees, so as to further enhance their willingness to stay in the village public health service jobs, to optimize the structure of community health services personnel.

For currently employed village public health service providers, the following six aspects can be adopted to improve their occupational environment, so as to improve their job satisfaction and reduce burnout and turnover. First, government departments of health will further optimize the coordination system of public health services between townships and villages. Scientific division of responsibilities, reasonable allocation of funds, effective training and guidance, standardized performance assessment system, reducing work load and other mechanisms make village public health service an attractive occupation (63). Second, we should enhance the public's respect for village public health service providers (71), give priority to solving their old-age security and living subsidies, solve their living difficulties, and attract and encourage excellent providers to stay at the grass-roots level. Third, equal pay for equal work should be achieved to ensure that the income of village public health service providers is no lower than that of township health center staff, and encourage them to do a good job in village public health services (72), especially in COVID-19 prevention and control. Fourth, let more new village doctors share public health tasks, so that existing public health service providers have time to attend training, so as to improve their knowledge and ability of epidemic prevention and control, and prevent the outbreak at the source. Fifth, through preferential policies, more highly educated and high-level public health personnel will be attracted to villages to provide guidance on public health services, replenish fresh blood, and improve the capacity and quality of rural public health service providers (73–76).

## Data availability statement

The original contributions presented in the study are included in the article/supplementary material, further inquiries can be directed to the corresponding author.

## Ethics statement

The studies involving human participants were reviewed and approved by Medical Ethics Committee of Jining Medical University. All participants provided oral consent before any data were collected. Oral consent was obtained instead of written consent, because the survey was anonymous and did not involve personal privacy.

## Author contributions

XZ, WZ, and LX conceptualized the idea. XZ performed the analyses and wrote the first draft of the manuscript. YZ, ZX, CW, and ZT checked and entered the data. ZD, XZ, and SG critically revised the manuscript. All the authors read and approved the final manuscript.

## Funding

This research was funded by Cultivation Project of National Natural (Social) Science Foundation of Jining Medical University, No. JYP2019SK02, which issued a call for proposals to undertake this evaluation. The funding body had representation on the evaluation steering group, but played no role in the collection and analysis of data, nor in the preparation of this paper. The authors' analysis and interpretation of the evidence are their own and not those of the funders.

## Conflict of interest

The authors declare that the research was conducted in the absence of any commercial or financial relationships that could be construed as a potential conflict of interest. The reviewer HL declared a shared affiliation with the author WZ to the handling editor at the time of review.

## Publisher's note

All claims expressed in this article are solely those of the authors and do not necessarily represent those of their affiliated organizations, or those of the publisher, the editors and the reviewers. Any product that may be evaluated in this article, or claim that may be made by its manufacturer, is not guaranteed or endorsed by the publisher.

## Abbreviations

COVID-19: Coronavirus Disease 2019
ANOVA: Analysis of Variance
MBI-GS: Maslach Burnout Scale general scale
SD: Standard Deviation
EFA: exploratory factor analysis
SEM: Structural Equation Modeling
AGFI: adjust goodness of fit index
GFI: goodness of fit index
NFI: normed fit index
CFI: comparative fit index
IFI: incremental
TLI: Tucker-Lewis's index
RMSEA: root mean square error of approximation
ANOVA: Analysis of Variance
IRB: Institutional Review Board
AVE: Average Variance Extracted
CR: Construct Reliability.
